# Non-Pharmacological Interventions Aimed at Changing the Gut Microbiota for Preventing the Progression of Diabetic Kidney Disease

**DOI:** 10.3390/nu17132112

**Published:** 2025-06-25

**Authors:** Małgorzata Szczuko, Anna Grudniewska, Anna Durma, Robert Małecki, Izabela Filipczyńska, Edward Franek, Karolina Kędzierska-Kapuza

**Affiliations:** 1 Department of Bromatology and Nutritional Diagnostics, Pomeranian Medical University in Szczecin, 71-460 Szczecin, Poland; 2 Lux Med Medical Centre in Warsaw, 49 Komitetu Obrony Robotników Street, 02-146 Warsaw, Poland; 3 Department of Diabetology and Internal Medicine, Medical University in Warsaw, Żwirki I Wigury 61 Street, 02-091 Warsaw, Poland; a__owczarczyk@wp.pl; 4 Department of Nephrology, Miedzyleski Specialist Hospital in Warsaw, Bursztynowa 2, Street, 04-749 Warsaw, Poland; robert.malecki@aol.pl; 5 Department of Reproduction and Gynecological Endocrinology, Medical University of Bialystok, 15-276 Bialystok, Poland; 6 Department of Internal Medicine, Endocrinology and Diabetology, National Medical Institute of the Ministry of Interior and Administration in Warsaw, 137 Wołoska Street, 02-507 Warsaw, Poland

**Keywords:** DKD, gut–kidney axis, gut microbiota, dysbiosis, nephroprotection, non-pharmacological interventions, dietary modifications, resistant starch, curcumin

## Abstract

Background: Diabetic kidney disease (DKD) affects 20–50% of individuals with diabetes. The aim of this review was to identify interventions that positively influence the gut microbiota in DKD. Methods: Identification of relevant studies was conducted via a systematic search of databases and registers using the PRISMA guidelines. This review examined the relevant literature published up to 5 January 2025, using a systematic search in PubMed and Scopus. The search was conducted with combinations of keywords including DKD and therapy, supplementation and gut microbiota, and supplementation or probiotics or fecal microbiota transplant. The initial search fielded 132 results from PubMed and 72 from Scopus, which was narrowed to 135 relevant studies. The exclusion criteria included non-English language studies, letters to the editor, and conference abstracts. Eligible studies were independently assessed by a minimum of three authors, with discrepancies resolved through consensus. Results: Gut microbiota-targeted interventions, including probiotics, synbiotics, and dietary modifications, show promise in modulating the gut microbiota, but evidence specific to DKD remains limited. Some natural food components such as polyphenols and anthocyanins modulate the composition of the gut microbiota translocation of uremic toxins, which slows down the progression of diabetic kidney disease. In animal models, fecal microbiota transplantation (FMT) has shown positive effects in regulating dysbiosis and beneficial effects in chronic kidney disease, but studies involving humans with DKD are insufficient. Conclusions: *Lactobacillus* and *Bifidobacterium* strains, administered at doses ranging from 0.6 to 90 billion CFU, may help lower urea and creatinine levels, but outcomes vary by disease stage, duration of therapy, and amount used. High-fiber diets (>10.1 g/1000 kcal/day) and supplements such as resistant starch and curcumin (400–1500 mg/day) may reduce uremic toxins through gut microbiota modulation and reduction in oxidative stress. The effect of sodium butyrate requires further human studies.

## 1. Introduction

Chronic kidney disease (CKD) affects more than 10% of adults worldwide [1]. While there are dozens of known causes of CKD, diabetes accompanies most cases of CKD and especially ESKD. The percentage of patients with end-stage kidney disease (ESKD) due to diabetes has been steadily increasing. ESKD, which requires renal replacement therapy, is highly invasive and represents a significant burden on national health funds [2,3]. The disease is associated with high mortality and poses a huge challenge to public health worldwide, urgently necessitating new strategies for protecting against DKD and slowing the progression of chronic kidney disease (CKD) in the course of diabetes mellitus (DM) [4,5]. Well-documented pharmacological interventions, such as inhibition of the renin–angiotensin–aldosterone system (RAAS), ACE inhibitors, and sodium–glucose cotransporter 2 (SGLT2) inhibitors, have been described in the literature [6,7,8]. The emergence of these new groups of drugs has initiated a significant paradigm shift in combating CKD and cardiovascular complications [9].

The composition and diversity of the gut microbiota are shaped by many endogenous and exogenous factors, with dietary factors playing a key role. For example, the Mediterranean diet, with a high fiber intake, leads to different microbial profiles compared to the Western diet, which is rich in saturated fats and simple sugars. In addition, age affects the gut microbiota through natural aging processes, which are often accompanied by a decrease in the diversity of commensal bacteria and an increase in susceptibility to dysbiosis [10]. Other important factors include antibiotic use, chronic diseases, immune status, and environmental factors, including sanitation and exposure to pathogens. Alpha diversity, which refers to the diversity of microorganisms within a single sample, is usually measured by species richness and evenness of distribution. Low alpha diversity may reflect a state of dysbiosis, characterized by reduced resistance to external factors, lower functional flexibility, and a higher risk of developing chronic diseases such as type 2 diabetes, inflammatory bowel disease, and chronic kidney disease. Beta diversity, which describes the variability in microbial composition between samples (e.g., between individuals), provides information about the influence of factors such as diet, environment, age, and health status on the gut microbiota. Low beta diversity in this case suggests homogenization of the gut microbiota, which may occur, for example, in populations with similar dietary habits or after exposure to antibiotics. Analysis of both types of diversity is a key element in the assessment of gut health and potential therapeutic strategies based on gut microbiota modulation [10,11]. The gut microbiota of patients with DKD is characterized by the expansion of the genera *Escherichia*, *Citrobacter*, and *Klebsiella* and the depletion of *Roseburia* [11].

Gut dysbiosis refers to natural changes in the gut microbiota caused by diet, medication use, immune system activity, and the condition of the intestinal mucosa. In patients with chronic kidney disease (CKD), urea retention occurs, leading to its diffusion into the intestinal lumen, which promotes the proliferation of ureolytic bacteria. This process contributes to microbial imbalance. Additional stress factors, such as oxidative stress, activation of bacteriophages, and production of bacteriocins, can intensify these changes. As a result, microbial diversity decreases and pathogenic bacterial groups begin to dominate [12]; there is a reduction in the number of bacteria such as *Lactobacillus* (lactic acid bacteria) and *Bifidobacterium (Bifidobacteria)*, along with an increase in populations of Gammaproteobacteria, Betaproteobacteria, and Enterobacteriaceae. Pathogens such as *Escherichia*, *Salmonella*, *Shigella*, and *Yersinia* are capable of inducing inflammation not only in the intestines but also in other organs due to the existence of the gut–liver axis. Through the portal vein system, the liver is exposed to bacterial endotoxins such as lipopolysaccharides (LPSs), which activate immune cells—particularly Kupffer cells—leading to the production of pro-inflammatory cytokines, including interleukin (IL-1β, IL-6, IL-8), tumor necrosis factor alpha (TNF-α), and the development of a systemic inflammatory response. This type of chronic, low-grade inflammation may play a significant role in the progression of both diabetes and nephropathy [13]. It has been established that indoxyl sulfate (IS) and p-cresyl sulfate (PCS), as well as phenols and ammonia produced by the gut microbiota, exert toxic effects through multiple mechanisms: induction of oxidative stress, activation of aryl hydrocarbon receptors (AhRs) and nuclear factor kappa B (NF-κB), increased expression of adhesion molecules, and stimulation of renal fibrosis via TGF-β and epithelial-to-mesenchymal transition (EMT) pathways. Studies indicate a strong correlation between IS and PCS concentrations and the rate of CKD progression, an increased risk of cardiovascular events (e.g., left ventricular hypertrophy and atherosclerosis), and chronic inflammation [13].

Many studies have highlighted the potential health benefits of incorporating so-called superfoods into the diets of patients with type 2 diabetes and chronic kidney disease (CKD) [14]. Foods such as berries, chia seeds, flaxseeds, green tea, broccoli, turmeric, and garlic are rich in bioactive compounds, including polyphenols, flavonoids, omega-3 fatty acids, fiber, and antioxidants, which exhibit anti-inflammatory, antioxidative, and glucose metabolism-modulating properties. In the context of CKD, certain superfoods may reduce oxidative stress, improve lipid profiles, and support gut microbiota balance, which may help slow disease progression and enhance overall metabolic health. However, due to dietary restrictions associated with electrolyte imbalances (e.g., hyperkalemia), the selection of appropriate foods should be individualized and supervised by a clinical dietitian. Despite promising findings, further randomized clinical trials are necessary to confirm the efficacy and safety of long-term superfood use in this patient population [15].

Understanding the mechanisms by which the gut microbiota exacerbates kidney damage in diabetes is crucial in the treatment of DKD and may result in the development of targeted DKD therapies [16]. All reports of non-pharmacological interventions improving the condition of the intestinal flora, and thus reducing deterioration of kidney function, are important from the point of view of patient care, which is why we decided to review the literature in this area [17,18].

## 2. Materials and Methods

**Figure 1:**
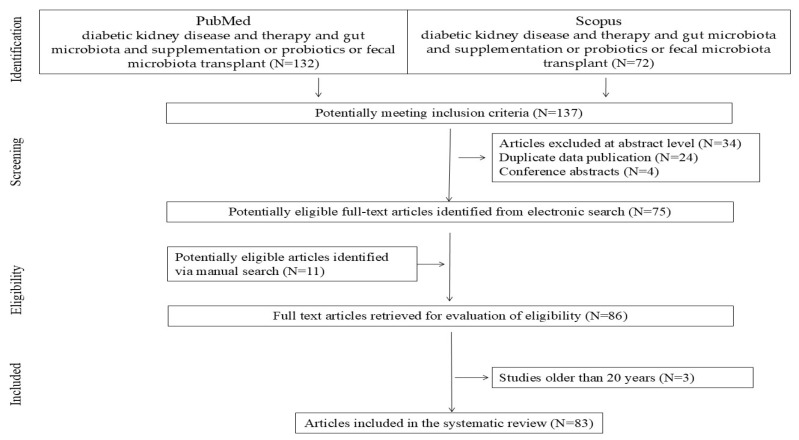
Research flow diagram.

This systematic review evaluates the above-mentioned topics and takes into account the literature published up to 5th January 2025. The reviewed studies were identified via a systematic search of databases and registers using the PRISMA guidelines. The search terms included DKD patient and dysbiosis/intervention, comparison, and time intervention. These terms were combined with prebiotics, synbiotics, gut microbiota, and superfoods. A systematic literature search was conducted using the PubMed and Scopus databases. The basic keywords were checked for combined terms of diabetic kidney disease and therapy, supplementation and gut microbiota, and supplementation or probiotics or fecal microbiota transplant (PubMed N = 132; Scopus N = 72). Firstly, article abstracts were analyzed and, if they contained significant information, the full text was analyzed. Studies that were not written in the English language and conference abstracts were excluded. Duplicate articles in both databases (n = 24), conference abstracts (n = 3), and unrelated abstracts (n = 35) were rejected. Finally, articles were added to supplement the content based on merit, i.e., articles identified via a manual search (n = 11). The PRISMA flow diagram is shown in Figure 1. Finally, the most up-to-date publications, published between 2004 and 2025, were selected. Data from these studies were extracted independently by one or two investigators and confirmed by a third investigator.

## 3. Superfoods

The term “superfoods” is used to describe foods that are considered to provide significant health benefits [12]. Typically, these “superfoods” are rich in specific nutrients, such as antioxidants, omega-3 fatty acids, and polyphenols. Research has shown these bioactive compounds to have beneficial effects on the body.

### 3.1. Polyphenols and Anthocyanins

Polyphenols can be divided into hydrolysable tannins (esters of gallic acid and saccharides) and compounds belonging to the phenylpropanoid class, such as flavonoids and lignans. The largest and most well-known group of polyphenolic compounds are flavonoids. Polyphenols have anti-inflammatory and antioxidant properties, which can have reno-protective effects and positively influence the gut microbiota [16]. Polyphenols are usually poorly absorbed in the intestines, but after biotransformation by the gut microbiota they undergo hydrolysis and absorption. The resulting compounds positively affect intestinal cells and promote the growth of specific types of bacteria [17]. Foods rich in bioactive compounds, such as turmeric, berries, cranberries, chocolate, propolis, beets, broccoli, garlic, cinnamon, coffee, and Brazil nuts, can modify the composition of the gut microbiota, modulate transcription factors related to inflammation, and reduce oxidative stress while positively affecting kidney function [18,19,20].

In turn, it has been shown that garlic (*Allium sativum* L.) can increase the diversity of the gut microbiota composition and restore reduced levels of *Lactobacillus* spp., *Bifidobacterium*, and *Prevotella* [21]. Metabolites produced by the gut microbiota from polyphenols provide numerous benefits for the intestinal barrier and immune system, enhancing antioxidant capacity. Phytochemicals produced by the gut microbiota are also important in modulating bacterial colonies [22,23].

Anthocyanins are a large group of plant pigments, classified as natural non-nutritive substances (NNSs) of plant origin, that are soluble in water [19]. These pigments are found in flowers, fruits, leaves, stems, and, less commonly, in roots and wood. Anthocyanins present in fruits and vegetables can modulate the composition of the gut microbiota, increasing the levels of *Bifidobacterium* spp., *Lactobacillus*, and *Enterococcus* spp., and enhancing the production of short-chain fatty acids (SCFAs), thereby improving the integrity of the intestinal barrier and modulating the immune system [24]. In particular, it has been shown that the consumption of blueberries, raspberries, cranberries, and strawberries can serve as a therapeutic strategy aimed at modulating the gut microbiota and reversing dysbiosis in chronic kidney disease [25]. Fiber-rich berries promote an increase in the diversity of the gut microbiota by affecting the amount of mucus-producing bacteria [26] and SCFA-producing bacteria. Moreover, these fruits can enhance the integrity of the intestinal barrier by increasing the expression of mRNA involved in the formation of tight junction proteins in the intestines, such as occludin, tight junction protein 1 (TJP1), and mucin [27]. They play a role in reducing the translocation of uremic toxins and lipopolysaccharides into the bloodstream, which slows the progression of CKD in patients.

### 3.2. Compounds Found in Certain Plants and Bee Products

Cinnamaldehyde, trans-cinnamaldehyde, cinnamic acid, and cinnamyl acetate, compounds found in cinnamon, can improve the integrity of the intestinal barrier and modulate the gut microbiota by inhibiting the growth of *Proteobacteria* and promoting *Bacteroidetes* [28].

Beetroot contains many biologically active phytochemicals, including betalains (e.g., betacyanins and betaxanthins), flavonoids, polyphenols, saponins, and inorganic nitrates (NO3); it is also a rich source of various minerals such as potassium, sodium, phosphorus, calcium, magnesium, copper, iron, zinc, and manganese [29]. Components of beetroot increase the diversity of the gut microbiota and the production of SCFAs [30].

Turmeric can also alter the composition of the gut microbiota, improve intestinal barrier permeability, and increase the catalytic activity of intestinal alkaline phosphatase. Curcumin, a bright yellow chemical produced by turmeric, has been approved as a food additive by the World Health Organization, the European Parliament, and the United States Food and Drug Administration. The meta-analysis in [31] suggests that the use of curcumin-containing supplements (400–1500 mg/day) is associated with significant reductions in serum hs-CRP and IL-6 levels in hemodialysis patients.

Propolis, or “bee glue”, is a resinous mixture produced by honeybees by mixing saliva and beeswax with pollen collected from plants. At a dose of 250 mg/day, propolis has significant anti-inflammatory properties, reducing cytokine levels [32]. Additionally, propolis acts as an anti-inflammatory substance by inhibiting and downregulating TLR4, MyD88, IRAK4, TRIF, NLRP inflammasomes, NF-κB, and associated pro-inflammatory cytokines such as IL-1β, IL-6, IFN-γ, and TNF-α. Propolis also reduces the migration of immune cells such as macrophages and neutrophils, likely by inhibiting the chemokines CXCL9 and CXCL10 [33,34].

Another study showed the therapeutic effects of plantaginis semen from the seeds of the Chinese herb plantaginis (*Plantago asiatica* L.) in DKD mice, particularly in mitigating renal lesions and glucose metabolism disorders. These effects may be attributed to the synergistic interaction of its bioactive components: iridoids, such as geniposidic acid; phenylethanol glycosides, such as acteoside; and guanidinium derivatives, including plantagoamidinic acid A. The participation of the SphK1-S1P signaling pathway in mediating these effects highlights a promising avenue for further research [35].

## 4. Sodium Butyrate

SCFAs act as a link and communicator between the gut microbiota and the immune system, playing a key role in maintaining balance between anti-inflammatory and pro-inflammatory responses, among other functions, by transmitting signals through free fatty acid receptors. A particular property of short-chain fatty acids is the induction of regulatory T cells (Treg), which occurs through the inhibition of the histone deacetylase enzyme. Butyric acid has the highest inhibitory potential, causing proliferation and increasing the functional capabilities of Treg cells [36]. It is mainly produced by bacteria of the genera *Clostridium*, *Eubacterium*, and *Fusobacterium* (e.g., *Clostridium butyricum* and *Eubacterium limosum*), with *Clostridium leptum*, *Roseburia* spp., *Faecalibacterium prausnitzii*, and *Coprococcus* spp. being particularly effective [37]. Intestinal dysbiosis in patients with diabetic kidney disease is particularly associated with a lower abundance of SCFA-producing bacteria belonging to *Ruminococcaceae*, *Lachnospiraceae*, and *Butyricicoccus* according to Li et al. [38]. In a study conducted by Cai et al., lower serum butyrate levels were noted in the group of patients with DKD (diabetic kidney disease), with a decrease in the number of SCFA-producing bacteria and low SCFA concentrations.

Current research suggests that butyrate also improves the course of acute kidney injury caused by ischemia, by inducing reperfusion [39]; contrast agent use [40]; gentamicin [41]; doxorubicin [42]; and hypertension [43].

The explanation of the molecular mechanisms of butyrate’s action in inhibiting the progression of DKD is multifaceted [44]. Firstly, as an agonist of the free fatty acid receptor family (GPR41, GPR43, and GPR109A), butyrate can exert anti-inflammatory effects through the signaling pathways of these receptors. GPR43 is expressed throughout the gastrointestinal tract, including on L-cells responsible for releasing peptide YY (PYY) and glucagon-like peptide-1 (GLP-1). It is believed to modulate body weight and reduce food intake, which positively affects diabetes control [45]. GPR41, on the other hand, is highly expressed on adipose tissue cells, where it induces the release of leptin—a hormone that significantly influences body weight—although other researchers report that this hormone acts indirectly through GPR43 [46,47]. Secondly, butyrate also acts as an epigenetic regulator in response to environmental stimuli or therapeutic modulation by inhibiting histone deacetylase (HDAC), stimulating miR-7a-5p, and regulating autophagy. Autophagy is a tightly regulated process that maintains cellular homeostasis [46]. Generally, SCFAs increase the expression of autophagy-related protein 7 in renal tubular epithelial cells, suggesting that SCFAs regulate autophagy in acute kidney injury [47]. Thirdly, butyrate-induced histone butyrylation has been shown to have protective effects on the kidneys in non-DKD nephropathies, creating a new perspective for designing clinical trials to investigate the therapeutic effects and molecular mechanism of butyrate in preventing and treating DKD.

## 5. Probiotics

An increase in urea and ammonia levels and rising pH promote the growth of aerobic bacteria, such as *Escherichia coli*, in the gastrointestinal tract and decrease the levels of anaerobic bacteria, such as *Bifidobacterium* and lactic acid bacteria. The consumption of *Bifidobacterium* restores the proper balance of bacteria in the gastrointestinal tract by limiting the proliferation of aerobic bacteria [48,49]. For this reason, *Bifidobacterium* and lactic acid bacteria can be used as probiotics, which are live bacterial cultures [50]. The bacteria use these substances as substrates for their metabolic processes. High doses of *Lactobacillus casei Shirota* (16 × 10^9 CFU) in diet led to a reduction in blood urea levels in 30 patients with CKD stages 3 and 4 over 8 weeks [51]. In a randomized double-blind, parallel group study conducted by Firouzi et al., it was proven that taking probiotics helps reduce urea levels, especially among overweight and obese individuals [52]; however, no statistical significance was observed in parameters such as potassium, sodium, and creatinine levels. Similarly, administering probiotics (a mix of *L. acidophilus KB27*, *B. longum KB31*, and *S. thermophilus KB19*) resulted in a reduction in blood urea nitrogen levels, although the reduction in creatinine and uric acid levels was not significant [53].

In patients with diabetic nephropathy (microalbuminuria in stages G1 and G2 according to KDIGO), the use of *L. plantarum A7* at a dose of 400 × 10^7^ CFU/day for 8 weeks reduced creatinine levels and improved the albumin-to-creatinine ratio (ACR) in urine [49]. Additionally, it reduced the level of IL-18, which is one of the markers of diabetic nephropathy progression [54]. It has been proven that supplementation with L. plantarum A7 probiotics in patients with diabetic nephropathy helps reduce the levels of cystatin C and progranulin (an inflammatory adipokine)—biomarkers of kidney function—thereby positively affecting kidney function [55].

The impact of probiotics on reducing uremic toxins was also assessed among patients undergoing hemodialysis. No benefits from probiotic supplementation were demonstrated. During the study, an increase in potassium levels and urea concentration was observed in patients using probiotics. It was proven that probiotic intervention did not significantly improve kidney parameters in the group of hemodialysis patients [56]. Similar conclusions were drawn by Natarajan et al. [57]. An intervention in the form of probiotic use (i.e., *S. thermophilus KB 19*, *L. acidophilus KB 27*, and *B. longum KB 31*) in a group of hemodialysis patients did not result in significant differences in uremic toxin levels, nor did it cause any adverse effects. The relatively small group of subjects (22 people) was a limitation of the study.

In a meta-analysis conducted by Dai et al. [58], the impact of probiotic use on kidney function among patients with diabetic kidney disease was assessed. Evaluating 10 randomized studies, it was found that probiotic supplementation intervention helps improve kidney parameters by delaying the increase in creatinine, cystatin C, and blood urea nitrogen levels, or by increasing the ACR index in urine. The meta-analysis showed that the reduction in creatinine is greater when the intervention period is longer than 8 weeks or the probiotic dose is less than 4 billion CFU/day [58], and that a greater reduction in blood urea nitrogen is observed with probiotic supplementation at a dose greater than 4 billion CFU/day. In a systematic review, Vlachou et al. demonstrated the beneficial effects of *Lactobacillus* and *Bifidobacterium* probiotics on patients with diabetic nephropathy [59]. The use of probiotics to improve kidney function still requires extensive research and in-depth analysis; however, it seems to be a safe procedure that can bring potential benefits (Table 1).

## 6. Synbiotics

Synbiotics are a combination of probiotics and prebiotics that work synergistically [60]. The combination of both substances enhances the effect compared to administering probiotics or prebiotics alone. Few studies have described the impact of synbiotics on kidney function in patients with diabetic kidney disease.

In a study conducted by Asemi et al., a 6-week supplementation of synbiotics containing 2.7 × 10^8^ CFU/day of *Lactobacillus sporogenes* and inulin, isomalt, sorbitol, and stevia in diabetic patients caused an increase in uric acid levels [61].

Rossi et al. evaluated an intervention administering synbiotics in patients with chronic kidney disease (among the subjects, 38% had diagnosed diabetic kidney disease, while the remaining patients had other etiologies of kidney disease) [62]. The subjects received prebiotics—inulin, fructooligosaccharides, and galactooligosaccharides—as well as probiotics—*Lactobacillus*, *Bifidobacteria*, and *Streptococcus*—for 6 weeks. The use of synbiotics resulted in a reduction in the levels of the uremic toxin p-cresyl sulfate; however, it did not affect the levels of indoxyl sulfate. Additionally, the administration of synbiotics positively influenced the modification of the gut microbiota by increasing the abundance of *Bifidobacterium* and decreasing *Ruminococcaceae*.

In another randomized study, a synbiotic consisting of a mixture of *Lactobacillus*, *Bifidobacterium*, and *Streptococcus* strains, combined with inulin and resistant tapioca starch, administered for 4 weeks to patients with CKD stages 3 and 4 according to KDIGO, reduced the total p-cresol levels [63]. This had an impact on cardiovascular risk, through the relationship between p-cresol levels and the occurrence of cardiovascular diseases [64]. However, the synbiotic administered in this study did not reduce gastrointestinal symptoms.

The use of synbiotics in chronic kidney disease does not always bring the expected benefits. In a study by McFarlane, after administering a synbiotic consisting of a mixture of *Bifidobacteria*, *Lactobacillus*, and *Streptococcus* and a prebiotic—resistant starch—for a year to patients with chronic kidney disease, an increase in creatinine levels and a decrease in eGFR were observed. In this study, 22% of the participants had diabetic nephropathy [65].

In another randomized study, after administering a synbiotic (a mix of *Lactobacillus*, *Bifidobacterium*, and *Streptococcus* strains combined with oligosaccharides) to patients with chronic kidney disease (98.5% of the participants had diabetes, which was the cause of nephropathy), no effect on eGFR and creatinine levels was observed, but a reduction in blood urea nitrogen levels was noted [66].

In a meta-analysis conducted by Firouzi evaluating the impact of probiotics, prebiotics, and synbiotics on kidney parameters, it was proven that the use of these supplements causes an insignificant reduction in eGFR and an increase in creatinine levels in the supplement group [67].

Due to the varying results of studies evaluating the impact of synbiotics on kidney function, the benefits of their use remain uncertain (Table 2). A study conducted by Guo et al. showed that there was an inverse association between probiotic/synbiotic/prebiotic or yogurt supplementation and prevalence of DKD in patients with diabetes mellitus type 2 [68].

## 7. Fecal Microbiota Transplantation

Fecal microbiota transplantation (FMT) is a method involving the introduction of stool from a healthy donor into the recipient’s gastrointestinal tract, aiming to alter the gut microbiota for normalization and to bring benefits to the recipient [69]. To perform the fecal transplant, the donor is screened for potential infections such as parasites, toxigenic C. difficile, and hepatotropic viruses. The stool can be introduced using various methods—colonoscopy, rectal enema, nasogastric tube, or capsules [70]. Dysbiosis present in kidney diseases has a harmful effect on the increase in uremic toxins in patients. Therefore, using fecal microbiota transplantation (FMT) as a method to modulate the gut microbiota in these patients promises potential health benefits. In animal studies, it was observed that transplanting gut microbiota to healthy individuals increased the levels of uremic toxins [71].

Many studies conducted on animal models have shown beneficial effects in terms of reducing the levels of uremic toxins, including p-cresyl sulfate and p-cresyl glucuronide [72], as well as decreasing the level of TNF-α in the ileum and ascending colon [73] after transplantation. Adverse changes were observed after fecal transplantation from CKD patients to healthy mice [74,75]. In a study conducted on patients with chronic kidney disease, diabetes, and/or hypertension, an intervention in the form of FMT administered in frozen capsules resulted in less progression of kidney disease 6 months after the intervention compared to the placebo group [76]. Relatively few studies have described the impact of FMT on the development of diabetic nephropathy. Nevertheless, studies have been conducted that have demonstrated the beneficial effects of FMT on chronic kidney disease of other etiologies. Therefore, it can be assumed that modifying the gut microbiota in diabetic patients will positively affect diabetic kidney disease. Zhou et al. described the case of a patient with membranous nephropathy and chronic diarrhea who underwent fecal transplantation from a healthy donor. The procedure was performed twice with a 28-day interval. After the fecal transplantation, an improvement in kidney parameters was observed, including a decrease in creatinine and urea levels and an increase in blood albumin levels [77]. Importantly, the fecal transplantation did not cause serious adverse effects. Zhao et al. described the case of two patients with IgA nephropathy who had previously undergone immunosuppressive treatment, which did not produce the desired effects [78]. They were given FMT and experienced partial remission of the disease, manifested by a reduction in the amount of protein in the 24 h urine collection and an increase in plasma albumin levels; there was no effect on blood creatinine and urea levels. Zhi et al. presented the case of a patient with focal segmental glomerulosclerosis, confirmed by kidney biopsy. After the implementation of glucocorticosteroids, there was an improvement in the kidney parameters, but after reducing the drug dose, creatinine levels increased. After FMT, the level of protein in the urine decreased, and no exacerbation of the disease was observed after reducing the glucocorticosteroid dose. An additional benefit was the observed decrease in triglyceride and cholesterol levels; FMT did not affect creatinine levels [79]. In summary, FMT can positively modulate the gut microbiota and thus influence the development of DKD [80] (Table 3).

## 8. Low-Protein Diet—Impact of Limiting Animal Protein on Gut Microbiota

Many uremic toxins are end products of the degradation of protein in the diet. Since protein absorption is impaired in both non-dialyzed and dialyzed patients with chronic kidney disease, increased amounts of undigested or unabsorbed proteins reach the colon, promoting the growth of proteolytic bacteria that produce uremic toxins. The fecal metabolome of hemodialysis patients can be clearly distinguished from that of healthy individuals of the same age but cannot be distinguished from the fecal metabolome of household members on a comparable diet, indicating the potential significance of diet in worsening kidney function in these patients. Hemodialysis (HD) patients are usually advised to limit potassium intake, which may lead to reduced consumption of fruits and vegetables and decreased dietary fiber intake. Intestinal transit time is prolonged in CKD, and constipation is common in dialysis patients, which is another factor contributing to dysbiosis and toxin absorption. While the prevalence of constipation ranges from 10 to 20% in healthy individuals, rates as high as 63% have been reported in HD patients and 29% in patients undergoing continuous ambulatory peritoneal dialysis (CAPD). The metabolic and hemodynamic effects of proteins are well known [81].

The amino acids phenylalanine and tyrosine are precursors of metabolites belonging to the classes of hippurans and phenols, respectively. Tryptophan participates in the formation of indoles, while ornithine, lysine, and arginine lead to the formation of polyamines. These metabolites cause harm the host’s health, including leading to progression of CKD [82]. Additionally, dietary protein increases the production of hydrogen sulfide (H2S) by gut bacteria. Undigested protein is a precursor of branched-chain SCFAs, which may have different roles to straight-chain SCFAs. SCFAs associated with dietary fiber are involved in regulating carbohydrate metabolism, while BSCFAs, such as isobutyric, isovaleric, and isocaproic acids, derived from the fermentation of undigested protein reaching the colon, do not have the same impact [83].

## 9. Research Directions

Personalization of gut microbiota, particularly in DKD, is recommended. Evaluation of the long-term effects of randomized intervention studies should include longer intervention periods (>8 weeks) and diverse patient groups to determine the effectiveness of probiotic and dietary therapies. Research into the molecular mechanisms of the action of SCFA, sodium butyrate, and receptor modulation (e.g., GPR41 and GPR43) in kidney protection could open new therapeutic possibilities. Studies on the long-term effects of fecal microbiota transplantation in DKD treatment could provide new evidence on the effectiveness of this intervention.

## 10. Conclusions and Limitations

Some natural food components, such as polyphenols, curcumin (400–1500 mg/day), and anthocyanins, modulate the composition of the gut microbiota translocation of uremic toxins, thereby slowing down the progression of diabetic kidney disease. A higher dietary fiber intake (>10.1 g/1000 kcal/day) reduces the risk of DKD.

Fecal microbiota transplantation (FMT) has been shown to have a positive impact on the regulation of dysbiosis and beneficial effects on chronic kidney disease in animal models; further studies involving humans with DKD are warranted.

Gut microbiota-targeted interventions, including probiotics, synbiotics, and dietary modifications, show promise in modulating the gut microbiota, but evidence specific to DKD remains limited. Dietary interventions such as high fiber intake and prebiotics have demonstrated benefits in reducing uremic toxins, but DKD-specific studies are scarce. Probiotics and synbiotics, particularly Lactobacillus and Bifidobacterium strains, administered at doses ranging from 0.6 to 90 billion CFU, may help lower urea and creatinine levels and improve kidney function, though results are inconsistent and disease-stage dependent. Studies have examined the effects of sodium butyrate on rats, but further study is required on humans.

## Figures and Tables

**Table 1:** Studies evaluating the use of probiotics in patients with chronic kidney disease.

Study	Probiotic Strain	Subjects and Duration of Supplementation	Results
Alatriste et al. [51]	*Lactobacillus casei Shirota* (16 × 10^9^ CFU)	30 patients with chronic kidney disease, stages 3 and 4, for 8 weeks	Lower ammonia levels in patients using probiotics.
Firouzi et al. 2015 [52]	Multi-strain preparation (*Lactobacillus acidophilus*, *Lactobacillus casei*, *Lactobacillus lactis*, *Bifidobacterium bifidum*, *Bifidobacterium longum*, and *Bifidobacterium infantis*) (6 × 10^10^ CFU)	136 people with type 2 diabetes for 12 weeks	Probiotic supplementation reduced urea levels, especially among overweight or obese individuals.
Fagundes et al. [53]	Mix *of L. acidophilus KB27*, *B. longum KB31*, and *S. thermophilus KB19* at a dose of 9 × 10^10^ CFU/day	46 patients with chronic kidney disease, stages 3 and 4, for 6 months	Probiotic supplementation reduced blood urea nitrogen levels. Additionally, an improvement in well-being was observed among those using probiotics.
Borges et al. [56]	*Streptococcus thermophilus*, *Lactobacillus acidophilus*, and *Bifidobacterium longum*, 90 billion CFU/day for 3 months	46 hemodialysis patients	Probiotic supplementation did not reduce uremic toxins or inflammatory markers.
Natarajan et al. [57]	*S. thermophilus KB 19*, *L. acidophilus KB 27*, and *B. longum KB 31* (90 billion CFU/day)	22 people with chronic kidney disease, stage 5	No statistically significant changes in uremic toxin levels were observed.

**Table 2:** Studies evaluating the use of synbiotics in patients with chronic kidney disease.

Study	Synbiotic	Subjects	Duration of Study	Result
Rossi et al. [62] 2016	Prebiotic—inulin, fructooligosaccharides, and galactooligosaccharides; probiotic—*Lactobacillus*, *Bifidobacteria*, and *Streptococcus*.	31 patients with chronic kidney disease	6 weeks	Reduction in p-cresyl sulfate levels; no effect on indoxyl sulfate levels. Beneficial modification of gut microbiota.
Guida et al., 2014 [63]	*Lactobacillus plantarum*, *2 × 10^9^ Lactobacillus casei subsp. rhamnosus*, *2 × 10^9^ Lactobacillus gasseri*, *1 × 10^9^ Bifidobacterium infantis*, *1 × 10^9^ Bifidobacterium longum*, *1 × 10^9^ Lactobacillus acidophilus*, *1 × 10^9^ Lactobacillus salivarius*, *1 × 10^9^ Lactobacillus sporogenes*, *and 5 × 10^9^ Streptococcus thermophilus +* prebiotic: inulin and 1.3 g of tapioca-resistant starch.	30 patients with CKD, stages 3–4	4 weeks	Reduction in p-cresol levels.
McFarlane et al. 2021 [65]	The prebiotic was 20 g/day of high-resistant starch fiber supplement and the probiotic component provided 4.5 × 10^11^ colony-forming units (CFU)/day of nine different strains from three different genera (*Bifidobacteria*, *Lactobacillus*, and *Streptococcus*).	68 patients with chronic kidney disease, stages 3–4	12 months	Modification of gut microbiota in patients using synbiotics—increase in Bifidobacterium and Blautia spp. Decrease in eGFR and increase in creatinine levels.
Dehghani et al. 2016 [66]	Prebiotic (fructooligosaccharide) + probiotic (seven strains across *Lactobacillus*, *Bifidobacteria*, and *Streptococcus*).	66 patients with CKD (stages 3 and 4)	6 weeks	Reduction in blood urea nitrogen levels after synbiotic use. No effect of synbiotic on creatinine levels.

**Table 3:** Studies evaluating fecal transplantation in patients with chronic kidney disease.

Animal Studies	Donor	Recipient	Result
Uchiyama et al., 2020 [71]	Mice suffering from chronic kidney disease	Healthy, germ-free mice	Development of insulin resistance and sarcopenia in recipients.
Barba et al. [75]	Healthy mice	Mice with chronic kidney disease	Regulation of dysbiosis in mice with chronic kidney disease. Additionally, improvement in glucose tolerance.
Bastos et al. [72]	Healthy mice	Mice with chronic kidney disease	Reduction in albuminuria, prevention of weight gain, and lower expression of TNF-α in the ileum and colon.
Shang et al. [73]	Healthy mice	Mice with DKD	Restoration of normal gut microbiota can alleviate the course of DKD.
**Human Studies**	**Donor**	**Recipient**	**Result**
Wang et al., 2020 [74]	Patients with end-stage renal disease (223 patients) or healthy patients; control group (69 patients)	Rodents with adenine-induced chronic kidney disease	Gut microbiota of patients with chronic kidney disease induces higher production of uremic toxins and greater kidney fibrosis in mice.
Zhou et al., 2021 [77]	14-year-old healthy male patient	Patient suffering from membranous nephropathy	Increase in serum albumin levels; decrease in creatinine and urea levels.
Zhao et al., 2021 [78]	Two healthy patients	Two patients suffering from IgA nephropathy	Lower protein excretion in 24 h urine collection; increase in blood albumin levels.
Zhi et al., 2022 [79]	Healthy patient	Patient suffering from focal segmental glomerulosclerosis	Reduction in urine protein levels; disease remission.
Arteaga-Muller et al., 2024 [76]	Healthy donors	Patients suffering from nephropathy secondary to diabetes and hypertension (13 in placebo group and 15 in FMT group); CKD in stages 2, 3, and 4	A higher number of patients (53.8% in the placebo group) experienced CKD progression compared to the FMT group (13.3%).

## Data Availability

Not applicable.
